# Human population structure detection via multilocus genotype clustering

**DOI:** 10.1186/1471-2156-8-34

**Published:** 2007-06-25

**Authors:** Xiaoyi Gao, Joshua Starmer

**Affiliations:** 1 Miami Institute for Human Genomics, University of Miami Miller School of Medicine, Miami, FL 33136, USA; 2 Department of Genetics, University of North Carolina at Chapel Hill, Chapel Hill, NC 27599, USA

## Abstract

**Background:**

We describe a hierarchical clustering algorithm for using Single Nucleotide Polymorphism (SNP) genetic data to assign individuals to populations. The method does not assume Hardy-Weinberg equilibrium and linkage equilibrium among loci in sample population individuals.

**Results:**

We show that the algorithm can assign sample individuals highly accurately to their corresponding ethnic groups in our tests using HapMap SNP data and it is also robust to admixed populations when tested with Perlegen SNP data. Moreover, it can detect fine-scale population structure as subtle as that between Chinese and Japanese by using genome-wide high-diversity SNP loci.

**Conclusion:**

The algorithm provides an alternative approach to the popular STRUCTURE program, especially for fine-scale population structure detection in genome-wide association studies. This is the first successful separation of Chinese and Japanese samples using random SNP loci with high statistical support.

## Background

Population structure analysis is important to genetic association studies and human evolutionary history investigations. Population structure may confound the population based genetic association studies, leading to false positive results and failure to detect true associations [1-4]. In studies of human evolution, populations are usually considered groups of interest and there has been a significant amount of work dedicated to learning about the relationships among modern populations [5-9].

Two major approaches have been proposed to detect population structure: distance-based clustering methods and model-based methods. Distance-based approaches utilize the proportion of allele sharing as distances between individuals and are not computationally demanding. Since some of the genetic information in the data is ignored, such as allele frequencies, critics have suggested that distance-based methods are not suitable for detecting fine population structure, even when many SNP loci are used [10]. Contrary to these assertions, in this paper we demonstrate the feasibility of using a distance-based method to detect fine population structures. Model-based methods use standard statistical methods to estimate population parameters, and usually assume Hardy-Weinberg equilibrium for each population. The inference may not be good in the presence of small sample sizes due to the inaccurate estimation of allele frequencies. Model-based inference also depends heavily on the modeling assumptions.

STRUCTURE is a popular model-based program using Markov chain Monte Carlo (MCMC) within a Bayesian framework [11]. However several issues in STRUCTURE have to be addressed carefully: missing data, the number of clusters, and the covergence of MCMC [12]. Moreover, STRUCTURE is computationally intensive. Recently, Purcell and Sham proposed to use the Expectation Maximization (EM) algorithm to detect population structure [13]. The EM algorithm is faster than STRUCTURE when many markers are involved in the calculation, but for large numbers of loci the computational load is still challenging. Both STRUCTURE and the EM algorithm assume that marker loci are in linkage equilibrium within subpopulations [11,13], which restricts the number of SNP loci that can be used. Even the improved version of STRUCTURE (version 2.0), which accounts for admixture Linkage Disequilibrium (LD), does not handle background LD well [14].

Many recent studies have been devoted to the human population structure analysis using DNA genetic markers. Bowcock et al. used 30 polymorphic microsatellites to construct trees of human inividuals that reflect their geographic origin with a neighbor-joining algorithm [6]. Mountain and Cavalli-Sforza also used a neighbor-joining algorithm to study human evolutionary history using an average of 75 RFLPs per individual [7]. Pritchard et al. pointed out that distance-based methods may be heavily dependent on the distance measure and graphical representation used, and may not be used for fine statistical inference [11]. Rosenberg et al. used STRUCTURE to analyze 377 autosomal microsatellite loci in 1056 individuals from 52 populations, and identified six main genetic clusters [8]. However, the solutions for the East Asia group were variable across runs when they tried to estimate population structure for the main regions. Turakulov and Easteal used Ward's algorithm, with a score matrix of Euclidean distances of allele sharing, for population structure analysis and concluded that the distance-clustering algorithm may not be used to detect fine-scale population structure [10]. Shriver et al. used 8,525 autosomal SNPs and a neighbor joining algorithm to infer population substructure in four populations: African-American, European-American, Chinese and Japanese [9]. They found a bifurcation between clusters of 10 Chinese and 10 Japanese individuals but with weak bootstrap support. Purcell and Sham used an EM algorithm on population structure analysis and validated the algorithm using simulated data but they did not show its efficacy with real data sets [13].

In this paper we describe a distance-based algorithm, AW-clust (Allele sharing distance and Ward's minimum variance hierarchical clustering), to assign individuals to populations based on multilocus genotype data. The method is different from previous distance-based approaches in many respects. Bowcock et al. used a neighbor-joining method to construct a human evolutionary tree [6], while we use Ward's minimum variance to estimate sub-clusters. Turakulov and Easteal used an Euclidean distance measure of allele mis-matches [10], while we used the allele sharing distance directly. Nakamura et al. required all markers to be unlinked and estimated the number of subpopulations, *K*, by cross-validation and the *k*-means algorithm in order to construct the hierarchical cluster [15], while the AW-clust method does not have these restrictions. The AW-clust method also differs in how it updates the distance matrix. Furthermore, the AW-clust method does not assume Hardy-Weinberg equilibrium and linkage equilibrium among loci in sample population individuals. There is also a conceptual difference in estimating *K*, the correct number of populations. We view this as a variable that need not be determined in advance.  Allowing *K *to vary gives researchers an opportunity to effectively define the resolution of their investigation in response to the particular problem they are addressing. If only rough distinctions need to be made between populations, the resulting tree can be examined at a high level. If the study requires highly refined distinctions, the tree can be examined at a low level. Once we had determined the number of SNP loci needed to differentiate populations, we used Tibshirani et al.'s gap statistic [16] to objectively verify *K*. 

We applied the AW-clust algorithm to two large human SNP data sets from the HapMap project [17] and Perlegen [18]. The algorithm accurately assigns HapMap sample individuals to the corresponding ethnic groups and it is also robust to admixed populations when tested on Perlegen data. Despite the suggestion that distance-clustering analysis may not detect fine-scale population structure [10,11], we have successfully differentiated Chinese and Japanese sample individuals using HapMap data. This is the first successful detection of fine-scale population structure as subtle as that between Chinese and Japanese with high statistical support using genome-wide random SNP loci. We anticipate that other closely related populations may be separated by similar approaches. Using a larger number of SNP markers, AW-clust has the power to accurately detect population structure and assign individuals to their ethnic group and shows that it is not be necessary to estimate allele frequencies or LD in order to differentiate populations.

## Results

### Results of simulation study

We found that both the number of SNP loci and the length of generations since isolation have a strong impact on the clustering results. When two populations had been isolated for time, *t *= 0.75 (scaled in terms of 2*N *generations), the mean ± standard deviation for the Classification Error Rates (CERs – see the Methods section for more details) were 0.01 ± 0.01, 0.05 ± 0.03 and 0.12 ± 0.05 using 200, 100 and 50 SNP loci respectively. For *t *= 0.05, the mean ± standard deviation for the CERs were 0.05 ± 0.03, 0.12 ± 0.05 and 0.22 ± 0.07 using 200, 100 and 50 SNP loci respectively. When the generations since isolation were relatively short, several hundred SNP loci may be insufficient to guarantee a low CER. When *t *= 0.015, the mean ± standard deviation for CER were 0.03 ± 0.02, 0.08 ± 0.03 and 0.17 ± 0.05 using 2000, 1000 and 500 SNP loci respectively. Therefore, the quality of separation is likely to be a function of *t*, the generations since isolation, and the number of SNP loci used. When *t *is long, a few loci are sufficient to guarantee a good separation. But when *t *is relatively short, a large number of SNP loci is required.

### Results from empirical data

**Figure 1:**
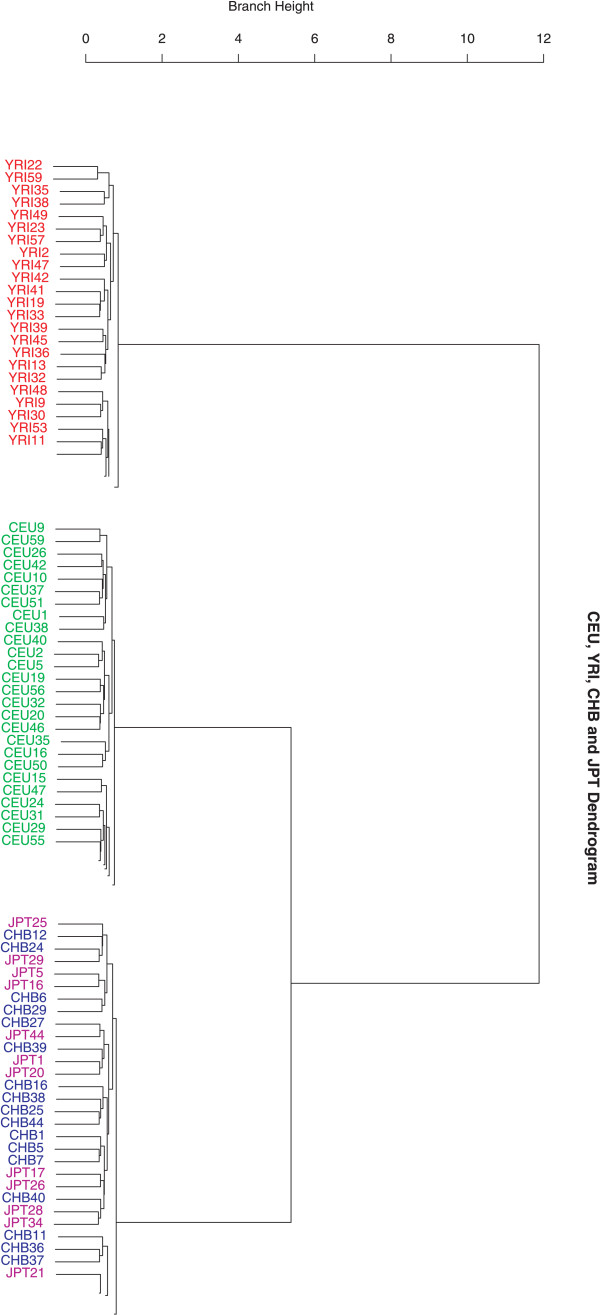
**A hierarchical cluster for the HapMap data with a sample of 200 SNPs**. A total of 209 unrelated individuals from four populations are shown: CEU (60), YRI (60) and CHB (45) + JPT (44). This figure shows a clustering result using 200 genome-wide random autosomal SNP loci. It is evident that YRI, CEU and CHB+JPT form three distinct clusters. Branch height represents dissimilarity. This figure shows the partial cluster, for the full image please see additional file 1.

We used the AW-clust algorithm to assign individuals to populations using HapMap data. A particular clustering result using 200 random SNP loci for the 209 unrelated sample individuals is plotted in Figure 1 (see also additional file 1). It is clear that there are three major clusters in the separation, with all YRIs forming one cluster, all CEUs forming another cluster, and all CHB+JPT forming the remaining cluster.

**Figure 2:**
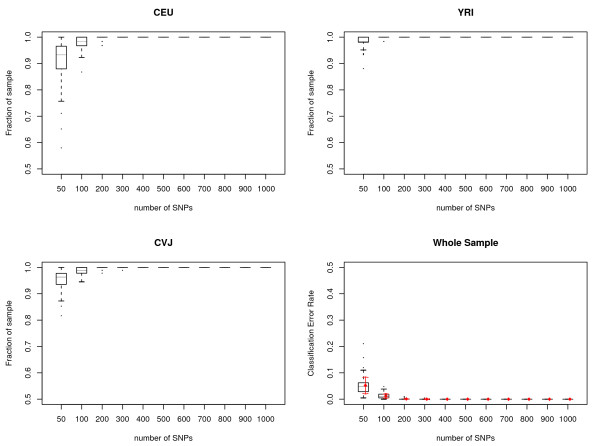
**The number of random SNP loci needed to correctly classify individuals in the HapMap data**. Boxplots show the statistics of predicted origin vs. known origin for CEU, YRI and CHB+JPT (CVJ) estimated with different numbers of SNP loci. Each dendrogram tree was cut at depth 2 to generate three clusters and predicted origin was assigned by the major population group represented in the cluster. Each number of SNPs was randomly sampled 100 times from 22 autosomal chromosomes. Horizontal lines are drawn at the 1st quartile, 3rd quartile and median and are connected to form the box. A vertical dashed line is drawn down from the 1st quartile to the most extreme data point within a distance of 1.5 interquartile range (IQR). A similar line is drawn up from the 3rd quartile. The ends of the vertical lines are indicated by short horizontal lines. Outliers are marked by dots. Red diamonds are the means of the classification error rate for the pooled whole sample for each number of SNP loci tested and red arrows are mean ± standard deviation.

What is the variation due to choice of SNP loci? What is the number of SNP loci needed to get a good clustering assignment? In order to answer these questions, we randomly selected 50–1,000 SNP loci from across the 22 autosomal chromosomes, and ran the AW-clust algorithm as described in the methods section. A dendrogram tree was cut at depth 2 to generate three clusters. We replicated each number of SNP loci tested 100 times to check the variation due to SNP sampling. The quality of clustering was plotted using boxplots in Figure 2. In the figure, we see that the quality of clustering is lowest and the variation is largest at 50 SNP loci. When 100 SNP loci were used, the mean accuracy of assignment is ~98.8% with a standard deviation of 0.01. Sample individuals could be assigned with ~100% accuracy when ≥ 200 SNPs were used. Clustering errors decreased with the increase of the number of SNP loci used. In the CEU, YRI and CHB+JPT (CVJ) sub-figures, all clustering plots with more than 400 SNP loci have 100% correct assignment.

**Figure 3:**
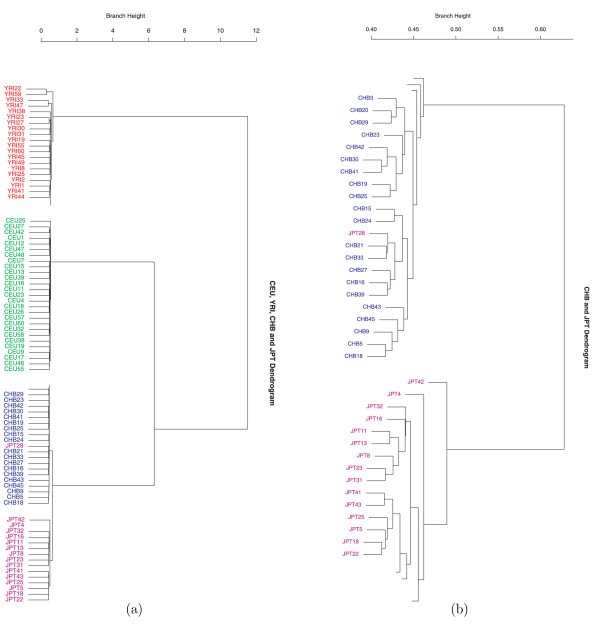
**Hierarchical clusters for the HapMap data with a sample of 20K SNPs**. (a) A total of 209 unrelated individuals from four populations are shown: CEU (60), YRI (60), CHB (45) and JPT (44). This figure shows a clustering result using 20K genome-wide random autosomal SNP loci. It is evident that YRI, CEU, CHB and JPT form four distinct clusters except for the misclassification of JPT28. Branch height represents dissimilarity. Notice that compared with YRI and CEU branch height, the CHB and JPT branch height is much shorter, representing that the genetic distance between these two populations is relatively close. This figure shows the partial cluster, for the full image please see additional file 2. (b) The magnified figure of CHB and JPT clusters in (a). This figure shows the partial cluster, for the full image please see additional file 3.

We then determined whether CHB and JPT samples could be differentiated. The cluster plot in Figure 3(a) (see also additional file 2) is based on one sample of 20,000 random SNP loci. We see four distinct clusters in the figure with all sample individuals clustered together according to their ethnic groups even though the branch length for CHB and JPT separation was much shorter compared with the branch length of CEU, YRI and CVJ. There was one misclassification: individual JPT28 was classified as CHB. Figure 3(b) (see also additional file 3) is the magnified figure of the CHB and JPT clusters in (a).

**Figure 4:**
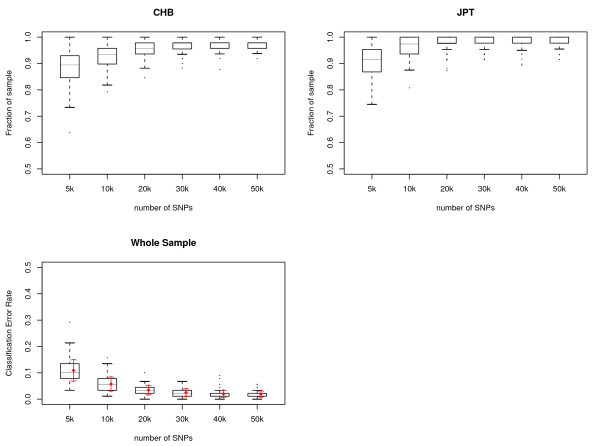
**The number of random SNP loci needed to correctly classify CHB and JPT from the HapMap data**. Boxplots show the statistics of predicted origin vs. known origin for CHB and JPT estimated with different numbers of SNP loci. Each number of SNPs was randomly sampled 100 times from 22 autosomal chromosomes. Horizontal lines are drawn at the 1st quartile, 3rd quartile and median and are connected to form the box. A vertical dashed line is drawn down from the 1st quartile to the most extreme data point within a distance of 1.5 interquartile range (IQR). A similar line is drawn up from the 3rd quartile. The ends of the vertical lines are indicated by short horizontal lines. Outliers are marked by dots. Red diamonds are the means of the classification error rate for the whole sample for each number of SNP loci tested and red arrows are mean ± standard deviation.

We also combined the 45 CHBs and 44 JPTs together and ran the AW-clust algorithm on the pooled sample. The variation due to SNP selection was also checked with 100 replications. The quality of clustering was plotted using boxplots in Figure 4. For the pooled whole sample, there was, on average, ~90% correct assignment when 5,000 SNP loci were used. We found that with 30,000 random SNPs we could get a mean accuracy of > 97% correct assignment with a standard deviation of 0.02. The number of random SNPs required to achieve this level of accuracy can be further reduced by eliminating less informative markers. After the pooled 89 individuals were separated into two clusters, we calculated the allele frequencies for each cluster and selected only the SNP loci with the absolute allele frequency differences > 0.15. This can reduce the number of SNPs more than 90%. We then re-ran the cluster analysis on the reduced SNP data set. The classification result was nearly as good as that from the full SNP data set. We did not get 100% correct assignment for CHB and JPT samples even when 500,000 random SNP loci were used. The misclassification is mainly from one individual, JPT28, which is likely to be misclassified as CHB.

**Figure 5:**
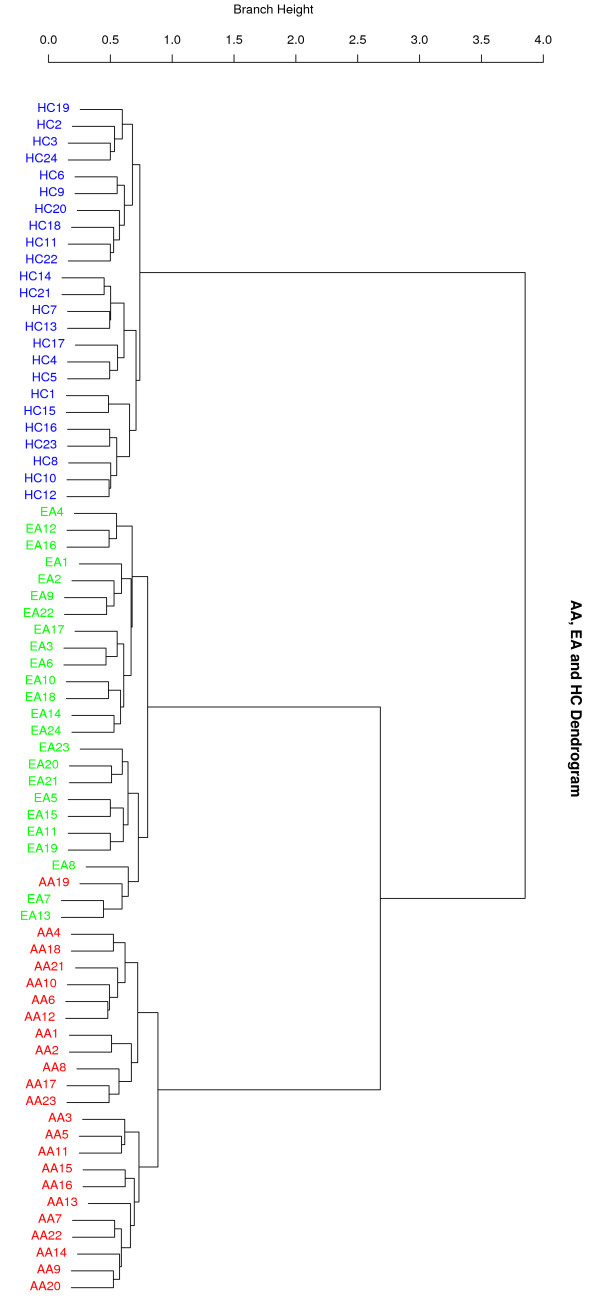
**A hierarchical cluster for the Perlegen data with a sample of 200 SNPs**. A total of 71 unrelated individuals from three populations are shown: AA (23), EA (24) and HC (24). This figure shows a clustering result using 200 genome-wide random autosomal SNP loci. It is evident that AA, EA and HC form three distinct clusters except for the misclassification of AA19. Branch height represents dissimilarity.

We also applied the AW-clust algorithm to the Perlegen SNP data set, which contains the admixed population African American (AA). We wanted to check the performance of the algorithm in the presence of admixed populations. A particular clustering result using 200 random SNP loci from the 71 unrelated sample individuals is shown in Figure 5. With the exception of the misclassification of AA19, it is clear that AA, EA and HC sample individuals form three discrete clusters. This shows that AW-clust can separate the admixed populations.

**Figure 6:**
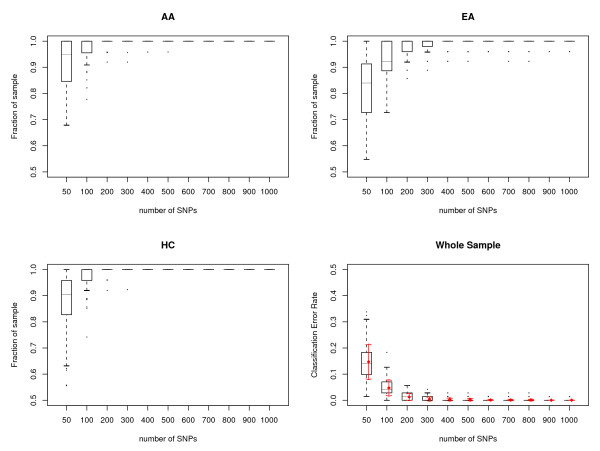
**The number of random SNP loci needed to correctly classify individuals in the Perlegen data**. Boxplots show the statistics of predicted origin vs. known origin for AA, EA and HC estimated with different numbers of SNP loci. Each dendrogram tree was cut at depth 2 to generate three clusters and predicted origin was assigned by the major population group represented in the cluster. For each number of SNPs, we randomly sampled 100 times from 22 autosomal chromosomes. Horizontal lines are drawn at the 1st quartile, 3rd quartile and median and are connected to form the box. A vertical dashed line is drawn down from the 1st quartile to the most extreme data point within a distance of 1.5 interquartile range (IQR). A similar line is drawn up from the 3rd quartile. The ends of the vertical lines are indicated by short horizontal lines. Outliers are marked by dots. Red diamonds are the means of the classification error rate for the sample for each number of SNP loci tested and red arrows are mean ± standard deviation.

The variation due to SNP sampling in the Perlegen SNP data set was also examined. We randomly selected 50–1,000 SNP loci from the 22 autosomal chromosomes, and ran the AW-clust algorithm to separate individuals into different clusters. A dendrogram tree was cut at depth 2 to generate three clusters. We replicated each number of SNP loci tested 100 times to check the variation due to SNP sampling. The quality of clustering was plotted by boxplots in Figure 6. The figure shows that the quality of clustering is lowest and the variation is largest with 50 SNP loci. When 100 random SNP loci were used, the mean accuracy of assignment is ~95.3% with a standard deviation of 0.032. Clustering errors decreased with the increase of the number of SNP loci used. When 200 SNPs were used in the clustering, the correct assignment had a mean of 98.7% and a standard deviation of 0.015. Increasing the number of SNP loci to 400 yielded a mean correct assignment of 99.7% with a standard deviation of 0.007. Only marginal improvements were seen when more than 400 SNPs were used. We also noticed that the rate of correct assignment increased much faster for AA and HC clusters than for EA cluster. AA and HC began to get close to 100% correct assignment when only 200 SNP loci were used, while EA required 400 SNP loci.

**Figure 7:**
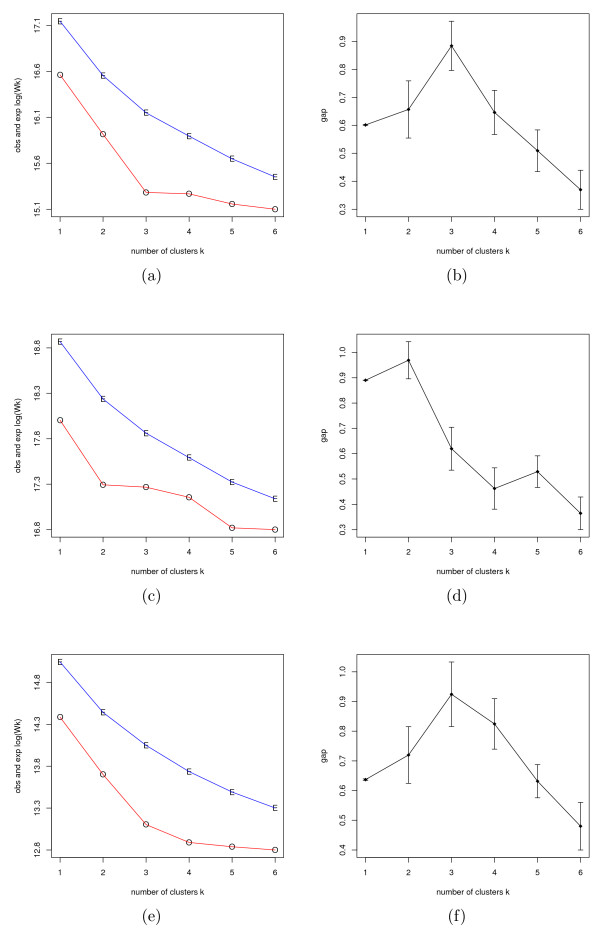
**Plots of the gap statistic**. The correct number of populations, *K*, was estimated via the gap statistic. In the left panel, the blue and red curves are the estimated expectation of log (*W*_*k*_) and the observed log (*W*_*k*_), respectively. The right panel is the gap statistic plot. The number of populations is set to range from 1 to 6. (a) and (b) correspond to the HapMap data, using 1,000 random genome-wide SNP loci. (c) and (d) correspond to the CHB and JPT data, using 30,000 random genome-wide SNP loci. (e) and (f) correspond to the Perlegen data, using 1,000 random genome-wide SNP loci. The inferred optimal *K *is the elbow point in the left panel, which is indicated by the maximizing gap on the right panel. It is clear that the gap statistic gives the optimal number of populations in each scenario as 3, 2, and 3, respectively.

In all empirical studies the estimation of correct number of populations, *K*, was identified from the major clusters in the hierarchical cluster plot. These values for *K *were then objectively verified using the gap statistic, in which three scenarios were considered. First, we considered three major populations in the HapMap data, CHB, YRI and CHB+JPT (CHB and JPT being indistinguishable), using 1,000 random genome-wide SNP loci. Second, only CHB and JPT individuals were included using 30,000 random SNP loci. Third, the three populations in the Perlegen data were considered using 1,000 random SNP loci. The gap statistic plots for each scenario are shown in Figure 7. The log (*W*_*k*_) estimations and gap curves are plotted in the left (Figure 7(a),(c) and 7(e)) and right panels (Figure 7(b),(d) and 7(f)), respectively. The observed and expected log (*W*_*k*_) are indicated in red and blue and marked by O and E separately for each *K *ranges from 1 to 6. The gap curved is plotted in the format of *Gap *(*k*) ± standard deviation of log (*W*_*k*_). The optimal *K *is the elbow point in the observed log (*W*_*k*_) plot, which corresponds to the maximizing point in the gap curve. From the plots, the estimated optimal *K *for each scenario is 3, 2 and 3, respectively. Multiple runs gave similar results. Therefore, the observation of *K *from the major clusters in the hierarchical cluster plot is reasonable and consistent with the gap statistic estimation.

## Discussion

To distinguish populations, the ideal loci are those that have an allele fixed in one population and absent in the other populations [19-21]. For the HapMap data, SNPs were sequenced in a small number of individuals, which means that SNPs with rare alleles are less likely to be discovered [22]. There are many measures for Ancestry Informative Markers (AIM), such as absolute allele frequency difference (*δ*), expected heterozygosity, *F *statistics (*F*_*ST*_), informativeness for assignment (*I*_*n*_) and informativeness for ancestry coefficients (*I*_*a*_) [20]. We did not use any AIM estimates for SNP selection in this study because we thought that the AIM estimates from one data set may not be safely applied to other data sets if the reference sample size was not large enough. Despite our SNP ascertainment procedures, we obtained very good results for population structure detection using HapMap and Perlegen data sets. The implication of this work is that it is possible to tackle population structure issues in genome-wide association studies with a large number of markers. Instead of a special marker selection procedure, the AW-clust algorithm uses a large number of random genome-wide SNPs to ensure a sufficient number of informative makers for inferences. Subpopulations can be identified, and then association tests are applied to each homogeneous group of individuals.

There are two key parts in the implementation of the AW-clust algorithm. The first part is the ASD distance matrix between all pairs of individuals. This distance was chosen because it can be shown using Balding and Nichols's DNA profile match probability theory [23] that the expected ASD between individuals from different subpopulations is always greater than that between individuals from the same subpopulation using SNP markers [24]. Thus, the within and between population distances may hardly overlap when many genome-wide random SNP loci are used. Therefore it is possible to differentiate populations from the half-matrix of pairwise distances without explicitly estimating allele frequencies for each sub-population. It is through the accumulated effect of many SNP loci that population structure can be identified. The second part in AW-clust is Ward's minimum variance algorithm, becuase inference of population structure based on ASD is likely to be reduced to contrasting group means and minimizing within-group variance of ASD. Ward's minimum variance appraoch is particularly suited to our problem, where the correct number of subpopulations is not known in advance, and we need to minimize the increase in the within-group ASD variance each time an individual is added to a cluster.

The advantage of our algorithm is that, relative to model-based methods, it is fast. Therefore, it can be applied to data sets with large numbers of individuals and SNPs, such as occur in genome-wide association studies. It took less than one minute on a desktop computer (P4 3.0G CPU with 1 GB memory) to separate CHB and JPT using 20K random SNP loci. Kruglyak (1999) estimated that approximately 500,000 uniformly distributed markers are required for genome-wide association studies. It would be valuable if we can make full use of these SNP markers in association studies for population structure protection. The AW-clust algorithm provides a possible solution to this scenerio. The AW-clust algorithm does not assume Hardy-Weinberg equilibrium or linkage equilibrium in the sample population individuals, no special marker selection criterion is required, and the algorithm is robust to relatively small sample sizes.

One drawback with distance-based methods is that the distance measure and the clustering algorithm are somewhat arbitrary. If another definition of distance or clustering method are used, the clustering results may change, which may be a reason, in addition to relatively small number of SNP loci used, why some authors did not see a separation of Chinese and Japanese sample individuals in previous studies [9,10]. A problem with Ward's minimum variance method is that it may or may not give the minimum possible error sum of squres over all possible sets of *K *clusters from the data units. However, Ward's solution is generally very good even if it is not optimal on this criterion [25]. We found that the classical multidimensional scaling (MDS) algorithm [26] can also be used to determine the ethnic clusters in the second stage of the AW-clust algorithm, which can be implemented in the standard statistical software package R using the *cmdscale *() function. Distance-based methods are criticized for being more suited to data exploration than to statistical inference [11]. However, we believe AW-clust could be effectively used as a first step in statistical inference. After using AW-clust to identify the major clusters, we can use Bayesian methods to calculate the posterior probabilities for individuals belonging to each different cluster. A general challenge for population structure analysis is to derive the correct number of subpopulations, *K*, and it is no different for the AW-clust algorithm. We view *K *as a variable instead of a fixed number and let researchers determine the most appropriate level of separation. For example, CHB and JPT are often grouped together for data analysis [17,27]. However, these two samples can be separated and fall in different clusters using AW-clust, as shown in the results. It is subjective whether we would like to treat them as one group or two groups and *K *should be defined to fit the researcher's interests and as the data permits. One possible alternative to the subjective definition of *K*, is to define it using the gap statistic. It should be noted, however, estimating *K *is still more art than science, and depends on many factors, such as population distance, number of individuals in each population, number of markers, random replicates and the method used.

It is easy to separate genetically distant ethnic groups, such as CEU (60), YRI (60) and CHB (45) + JPT (44) in the HapMap data, AA (23), EA (24) and HC (24) in the Perlegen data (sample size is in parenthesis). Both STRUCTURE (version 2.1) and the AW-clust algorithm gave very good classification in these situations using several hundred of random SNP loci. But the burn-in period and number of iterations in STRUCTURE may not be easily decided. Different authors used different settings [8,11-13,20,21,28]. In our tests, it seems that these settings depend on the sample size, the number of loci, and the genetic distances among populations in order to get the correct number of populations, *K*. STRUCTURE easily found the correct *K *= 3 with 5,000 burn-in followed by 1,000 iterations when we tested it on CEU (60), YRI (60) and CHB (45) + JPT (44) using 200 random SNP loci. However, when we reduced the CHB sample size to 10 (CEU (60), YRI (60) and CHB (10)), we needed to set 10,000 burn-in and 10,000 iterations for STRUCTURE in order to get the correct *K *using 200 random SNP loci. Another challenging situation for STRUCTURE is fine-scale population structure detection, such as that between Chinese and Japanese. STRUCTURE assumes that marker loci are in linkage equilibrium within subpopulations [11], which theoretically puts a restriction on the number of SNP loci that we can use from the human genome data. Even when we ignored this assumption and used 10,000 random SNPs in STRUCTURE, it did not separate CHB (60), YRI (60), CHB (45) and JPT (44) simultaneously since CHB and JPT were predicted to be in one cluster. This contrasts with the AW-clust algorithm, which separated CHB and JPT into two different clusters. When we chose to run STRUCTURE on the pooled CHB (45) and JPT (44) using 5,000 random SNP loci with a 5,000 burn-in period followed by 1,000 iterations, STRUCTURE identified two major clusters from the posterior probabilities of *K*. But when we reduced the sample size for JPT from 44 to 20, STRUCTURE failed to identify the correct *K*, even with 10,000 iterations after a burn-in period of 10,000 were used. The AW-clust identified two major clusters for CHB and JPT in the above situations. Neither of the two methods worked well when we reduced the sample size for JPT from 44 to 10 individuals using 5,000 random SNP loci. However, AW-clust created two discrete clusters for CHB (45) and JPT (10) when we increased the number of SNP loci to 20,000. STRUCTURE may or may not find the correct number of clusters, *K*, in the fine scale population structure situations using random SNP loci especially when a population has a relatively small sample size, such as 5 or 10 individuals, in addition to the considerable computing time consumed. If accurate AIMs are available, a likelihood approach should work well when predicting an individual's ethnicity [29].

STRUCTURE usually requires multiple runs to check the convergence of MCMC (STRUCTURE manual) which requires substantial computing time when a large number of individuals and SNP loci are used. The admixture proportions for each individual, *Q*, is considered an advantage of STRUCTURE. However, in our test on the Perlgen data set (AA is an admixture population), the inferred ancestry of individuals, Q^
 MathType@MTEF@5@5@+=feaafiart1ev1aaatCvAUfKttLearuWrP9MDH5MBPbIqV92AaeXatLxBI9gBaebbnrfifHhDYfgasaacH8akY=wiFfYdH8Gipec8Eeeu0xXdbba9frFj0=OqFfea0dXdd9vqai=hGuQ8kuc9pgc9s8qqaq=dirpe0xb9q8qiLsFr0=vr0=vr0dc8meaabaqaciaacaGaaeqabaqabeGadaaakeaacuWGrbqugaqcaaaa@2DE7@, may be sensitive to the number of SNP loci and sample sizes used. For example, we ran STRUCTURE with 50,000 burn-in followed by 50,000 iterations on AA(23), EA(24) and HC(24) using 5,000 random SNP loci. All EA individuals were predicted to have ~10% membership with HC, while most of the predicted HC membership in EA individuals went away when we used only 1,000 random SNP loci. With 50,000 burn-in and 50,000 iterations, nearly half of the AA individuals were predicted to have some EA membership, the majority of the EA individuals were predicted nearly pure EA membership, and most of the HC individuals were predicted nearly pure HC membership for the data AA(23), EA(24) and HC(24) using 1000 random SNP loci. Most of the AA individuals' EA membership either disappeared or was predicted to be much smaller when we reduced the sample size of AA from 23 to 5 and kept EA(24) and HC(24) using 1,000 random SNP loci. But when the sample size setting is AA(23), EA(5) and HC(24), EA individuals showed ~20% membership with HC. The apparent advantage of the AW-clust algorithm over STRUCTURE is for fine scale population structure detection with small sample sizes since a large number of SNP loci can be used and a relatively short computing time is required. The correct number of populations may be easily identified from the major clusters in the hierarchical plot rather than through multiple runs of rough estimation from MCMC posterior probabilities of *K*, which depend on many factors, such as the length of burn-in, iterations, convergence, and number of populations in the sample.

## Conclusion

In summary, the AW-clust algorithm provides efficient calculation and visually appealing results. It can produce highly accurate clustering and assign individuals correctly to populations. This algorithm successfully differentiated the CEU, YRI, and CHB+JPT sample individuals in the HapMap data set. It is also robust to the admixed population AA in the Perlegen data set, which covers AA, EA and HC sample individuals. Moreover, it can detect fine-scale population structure as subtle as that between CHB and JPT. We anticipate that other closely related populations can also be separated by similar approaches. Our method combined with SNP markers has considerable power in population structure analysis and it is not necessary to estimate allele frequencies in order to differentiate populations.

## Methods

### Data

SNP data were drawn from the HapMap project Phase I and the Perlegen SNP data sets. In the HapMap Phase I SNP data, about 1.1 million SNPs were genotyped genome-wide from 269 individuals from four ethnic populations: 90 individuals (30 trios) from Yoruba in Ibadan, Nigeria (YRI), 90 individuals (30 trios) from CEPH in Utah residents with ancestry from northern and western Europe (CEU), 45 unrelated Han Chinese from Beijing, China (CHB), and 44 Japanese from Tokyo, Japan (JPT), among which there are 209 unrelated individuals [17,30]. The Perlegen SNP data set is denser and it contains about 1.6 million SNPs in 71 unrelated individuals from three ancestry populations: 23 African Americans (AA), 24 European Americans (EA) and 24 Han Chinese (HC) from the Los Angeles area [18]. These two large SNP data sets offer high quality and rich density SNP genotypes for the population structure analysis of these ethnic groups. In this study, SNP loci were selected by random sampling from 22 autosomal chromosomes and only unrelated individuals were used.

### AW-clust algorithm

The AW-clust (Allele sharing distance and Ward's minimum variance hierarchical clustering) method consists of two stages. In the first stage, a distance matrix between all pairs of individuals is constructed. The distance between individuals *i *and *j *was defined as

Dij=1L∑l=1Ldij(l),
 MathType@MTEF@5@5@+=feaafiart1ev1aaatCvAUfKttLearuWrP9MDH5MBPbIqV92AaeXatLxBI9gBaebbnrfifHhDYfgasaacH8akY=wiFfYdH8Gipec8Eeeu0xXdbba9frFj0=OqFfea0dXdd9vqai=hGuQ8kuc9pgc9s8qqaq=dirpe0xb9q8qiLsFr0=vr0=vr0dc8meaabaqaciaacaGaaeqabaqabeGadaaakeaacqWGebardaWgaaWcbaGaemyAaKMaemOAaOgabeaakiabg2da9maalaaabaGaeGymaedabaGaemitaWeaamaaqahabaGaemizaq2aaSbaaSqaaiabdMgaPjabdQgaQjabcIcaOiabdYgaSjabcMcaPaqabaaabaGaemiBaWMaeyypa0JaeGymaedabaGaemitaWeaniabggHiLdGccqGGSaalaaa@42B3@

where

dij(l)={0,if individual i and j have two alleles in common at the l th locus,1,if individual i and j have only a single allele in common at the l th locus,2,if individual i and j have no allele in common at the l th locus,
 MathType@MTEF@5@5@+=feaafiart1ev1aaatCvAUfKttLearuWrP9MDH5MBPbIqV92AaeXatLxBI9gBaebbnrfifHhDYfgasaacH8akY=wiFfYdH8Gipec8Eeeu0xXdbba9frFj0=OqFfea0dXdd9vqai=hGuQ8kuc9pgc9s8qqaq=dirpe0xb9q8qiLsFr0=vr0=vr0dc8meaabaqaciaacaGaaeqabaqabeGadaaakeaacqWGKbazdaWgaaWcbaGaemyAaKMaemOAaOMaeiikaGIaemiBaWMaeiykaKcabeaakiabg2da9maaceqabaqbaeaabmGaaaqaaiabicdaWiabcYcaSaqaaiabbMgaPjabbAgaMjabbccaGiabbMgaPjabb6gaUjabbsgaKjabbMgaPjabbAha2jabbMgaPjabbsgaKjabbwha1jabbggaHjabbYgaSjabbccaGiabdMgaPjabbccaGiabbggaHjabb6gaUjabbsgaKjabbccaGiabdQgaQjabbccaGiabbIgaOjabbggaHjabbAha2jabbwgaLjabbccaGiabbsha0jabbEha3jabb+gaVjabbccaGiabbggaHjabbYgaSjabbYgaSjabbwgaLjabbYgaSjabbwgaLjabbohaZjabbccaGiabbMgaPjabb6gaUjabbccaGiabbogaJjabb+gaVjabb2gaTjabb2gaTjabb+gaVjabb6gaUjabbccaGiabbggaHjabbsha0jabbccaGiabbsha0jabbIgaOjabbwgaLjabbccaGiabdYgaSjabbccaGiabbsha0jabbIgaOjabbccaGiabbYgaSjabb+gaVjabbogaJjabbwha1jabbohaZjabcYcaSaqaaiabigdaXiabcYcaSaqaaiabbMgaPjabbAgaMjabbccaGiabbMgaPjabb6gaUjabbsgaKjabbMgaPjabbAha2jabbMgaPjabbsgaKjabbwha1jabbggaHjabbYgaSjabbccaGiabdMgaPjabbccaGiabbggaHjabb6gaUjabbsgaKjabbccaGiabdQgaQjabbccaGiabbIgaOjabbggaHjabbAha2jabbwgaLjabbccaGiabb+gaVjabb6gaUjabbYgaSjabbMha5jabbccaGiabbggaHjabbccaGiabbohaZjabbMgaPjabb6gaUjabbEgaNjabbYgaSjabbwgaLjabbccaGiabbggaHjabbYgaSjabbYgaSjabbwgaLjabbYgaSjabbwgaLjabbccaGiabbMgaPjabb6gaUjabbccaGiabbogaJjabb+gaVjabb2gaTjabb2gaTjabb+gaVjabb6gaUjabbccaGiabbggaHjabbsha0jabbccaGiabbsha0jabbIgaOjabbwgaLjabbccaGiabdYgaSjabbccaGiabbsha0jabbIgaOjabbccaGiabbYgaSjabb+gaVjabbogaJjabbwha1jabbohaZjabcYcaSaqaaiabikdaYiabcYcaSaqaaiabbMgaPjabbAgaMjabbccaGiabbMgaPjabb6gaUjabbsgaKjabbMgaPjabbAha2jabbMgaPjabbsgaKjabbwha1jabbggaHjabbYgaSjabbccaGiabdMgaPjabbccaGiabbggaHjabb6gaUjabbsgaKjabbccaGiabdQgaQjabbccaGiabbIgaOjabbggaHjabbAha2jabbwgaLjabbccaGiabb6gaUjabb+gaVjabbccaGiabbggaHjabbYgaSjabbYgaSjabbwgaLjabbYgaSjabbwgaLjabbccaGiabbMgaPjabb6gaUjabbccaGiabbogaJjabb+gaVjabb2gaTjabb2gaTjabb+gaVjabb6gaUjabbccaGiabbggaHjabbsha0jabbccaGiabbsha0jabbIgaOjabbwgaLjabbccaGiabdYgaSjabbccaGiabbsha0jabbIgaOjabbccaGiabbYgaSjabb+gaVjabbogaJjabbwha1jabbohaZjabcYcaSaaaaiaawUhaaaaa@3BCA@

and *L *is the number of SNP loci used. This, and similar pair-wise distance measures were used by Bowcock et al. [6], Mountain and Cavalli-Sforza [7] and Nakamura et al. [15].

The second stage uses hierarchical clustering to determine ethnic categories. We inferred clusters of individuals from the distance matrix according to Ward's minimum variance algorithm [31,32]. Here we briefly review the algorithm. In the initial step, each cluster contains one individual. At each step, the algorithm merges the two groups that will result in the smallest increase in the value of within-cluster variance. The pair is then joined and the number of clusters reduced by one. The clustering process continues until one cluster contains all individuals. Therefore, the within-cluster variance takes the minimal increase at each fusion. The cluster variance increases nonlinearly as the clustering process builds up [33], which clearly indicates where groups separate from each other. In this paper, Ward's minimum variance algorithm was implemented with the standard statistical software, R, using the function *hclust *() [34].

### Choice and validation of *K*

We regard the correct number of populations, *K*, as a variable instead of a fixed number that depends on the analysis rather than being an intrinsic biological variable. It depends on the level of population structure that we aim to detect and the number of SNP loci used. For example, we may not be able to separate the CHB and JPT individuals with a small number of random SNP loci but with more markers these two populations are differentiable. The *K *in this paper is identified from the number of major clusters in the hierarchical cluster plot.

Once we determined number of SNP loci needed to differentiate populations, we then confirmed the correct number of populations, *K*, using the gap statistic [16]. If *K *ranges from 1 to *k*, the gap statistic selects the optimal *k *such that log (*W*_*k*_), where *W*_*k *_is the pooled within-cluster sum of squares, is farthest below its null reference distribution curve. The gap statistic is defined as *Gap *(*k*) = E* {log (*W*_*k*_)} - log (*W*_*k*_), where E* denotes the expectation from the null reference distribution, in which a uniform distribution was used.

### Clustering quality

A sample was considered correctly assigned if the cluster with the major fraction of ancestry was the same as the ethnic group to which that individual was known to belong [21]. The clustering quality with a given number of SNP loci for each group was measured by Fraction Of Sample (FOS), which is defined as the ratio of the number of individuals of the major group in the cluster (known origin) over all the individuals assigned to the cluster (predicted origin) [10]. We also calculated the Classification Error Rate (CER) for the pooled sample as the proportion of incorrectly assigned individuals in the whole data set. The accuracy of assignment for the pooled sample is defined as 1-CER. Random variation due to SNP sampling was examined by a re-sampling method. SNPs were randomly chosen without replacement from all the SNPs used to re-run the analysis. In each case 100 replicates were performed.

### Simulation study

To evaluate the performance of the clustering algorithm in situations where the classifications are known, we simulated SNP data using the standard coalescent approach [35]. We considered samples of *n *= 50 individuals drawn from two random mating populations, each of size *N *= 10, 000, that had split from a single ancestral population at *t *generations in the past. The mutation rate at each simulated locus was assumed to be negligible. Given the SNP mutation rate is about 10^-8 ^per locus per generation [36], this assumption is likely to be valid. Genotypes for *n *individuals from each population were generated by random pairing of 2*n *alleles. We retained only those loci for which polymorphisms remained in the two populations to the current sampling time, *t *= 0.

We estimated the pair-wise distance between individuals from the set of genotypes for the 2*n *simulated individuals. Then we used the Ward's minium variance method to construct a tree from these distances. The quality of separation was evaluated by CER for the 2*n *individuals. For each set of parameters, we performed 100 simulations.

## Authors' contributions

XG designed the study, performed the statistical analysis, and drafted the manuscript. JS helped to draft the manuscript. All authors read and approved the final manuscript.

## Supplementary Material

Additional File 1 This figure shows the full image of Figure 1. Click here for file

Additional File 2 This figure shows the full image of Figure 3 (a). Click here for file

Additional File 3 This figure shows the full image of Figure 3 (b). Click here for file
