# Supplementary figures and images for: Human population structure detection via multilocus genotype clustering
Source: BMC Genet. 2007 Jun 25;8:34. doi: 10.1186/1471-2156-8-34 (PMC1934381; doi:10.1186/1471-2156-8-34)

Branch Height

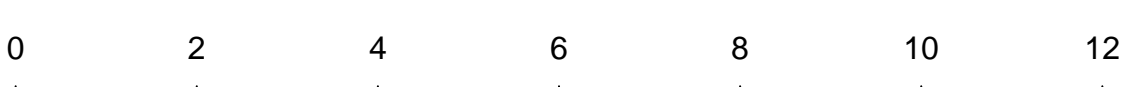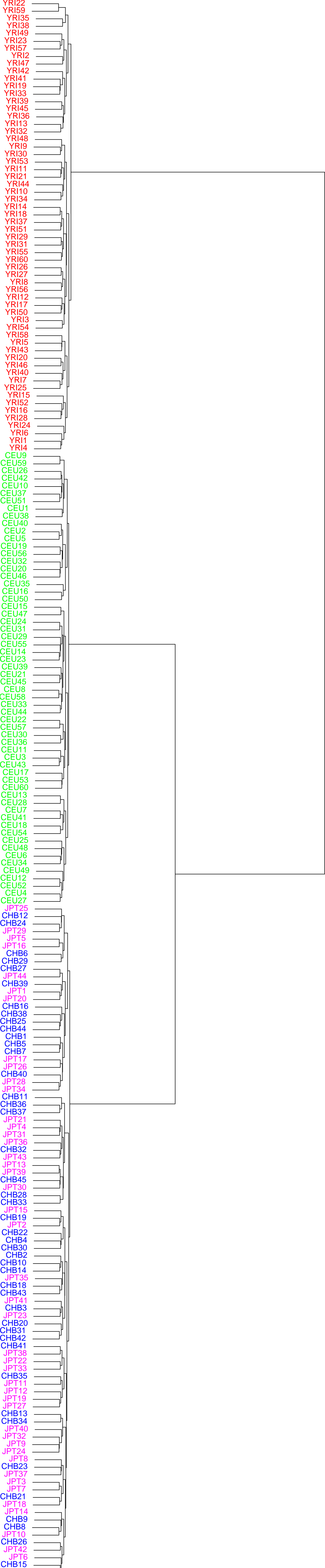

CEU, YRI, CHB and JPT Dendrogram

Supplement: Additional File 1 — This figure shows the full image of Figure 1. [file 1471-2156-8-34-S1.pdf]

Branch Height

0.40 0.45 0.50 0.55 0.60

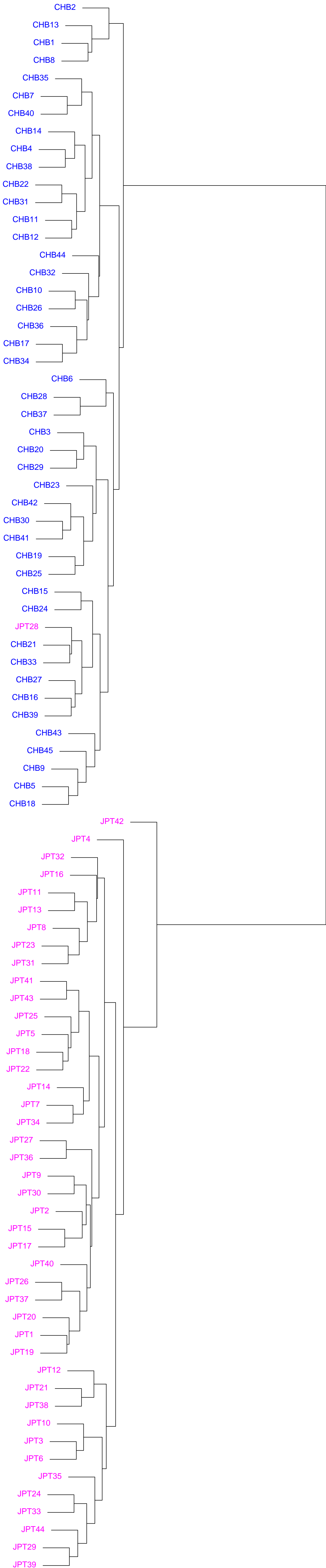

CHB and JPT Dendrogram

Supplement: Additional File 3 — This figure shows the full image of Figure 3 (b). [file 1471-2156-8-34-S3.pdf]
