# Supplementary material for: Human population structure detection via multilocus genotype clustering
Source: BMC Genet. 2007 Jun 25;8:34. doi: 10.1186/1471-2156-8-34 (PMC1934381; doi:10.1186/1471-2156-8-34)
Supplement: Additional File 2 — This figure shows the full image of Figure 3 (a). [file 1471-2156-8-34-S2.pdf]

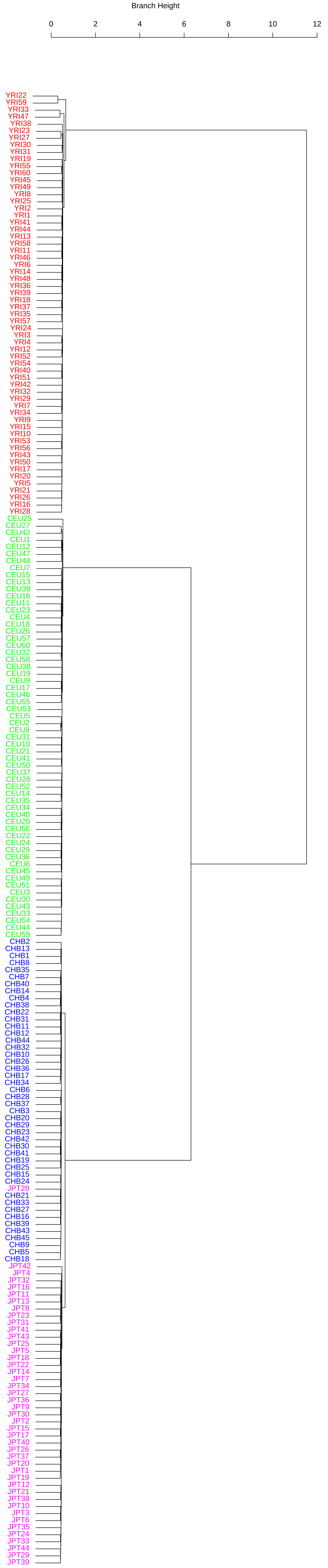

CEU, YRI, CHB and JPT Dendrogram
